# Charge-Transfer Spectroscopy of Ag^+^(Benzene) and Ag^+^(Toluene)

**DOI:** 10.1021/acs.jpca.3c01790

**Published:** 2023-05-25

**Authors:** Jason E. Colley, Dylan S. Orr, Michael A. Duncan

**Affiliations:** Department of Chemistry, University of Georgia, Athens, Georgia 30602, United States

## Abstract

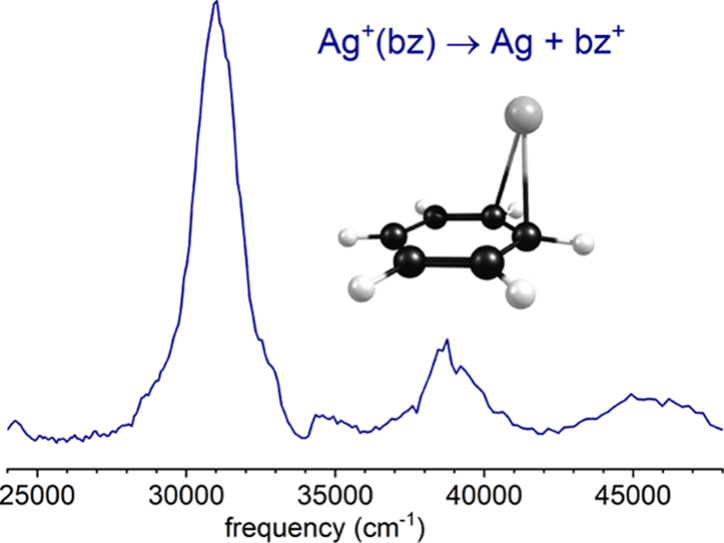

Gas-phase ion–molecule complexes of silver cation with benzene or toluene are produced via laser vaporization in a pulsed supersonic expansion. These ions are mass-selected and photodissociated with tunable UV–visible lasers. In both cases, photodissociation produces the organic cation as the only fragment via a metal-to-ligand charge-transfer process. The wavelength dependence of the photodissociation produces electronic spectra of the charge-transfer process. Broad structureless spectra result from excitation to the repulsive wall of the charge-transfer excited states. Additional transitions are detected correlating to the forbidden ^1^S → ^1^D silver cation-based atomic resonance and to the HOMO–LUMO excitation on the benzene or toluene ligand. Transitions to these states produce the same molecular cation photofragments produced in the charge-transfer transitions, indicating an unanticipated excited-state curve-crossing mechanism. Spectra measured for these ions are compared to those for ions tagged with argon atoms. The presence of argon causes a significant shift on the energetic positions of these electronic transitions for both Ag^+^(benzene) and Ag^+^(toluene).

## Introduction

Cation−π bonding has far-reaching applications in chemistry and biology,^1−8^ and it is essential to understand the structures and energetics of these interactions. Cation−π interactions mediate enzyme–substrate binding, antigen–antibody recognition, and protein folding. Similar bonding influences organometallic catalysis, including the binding of “single site”-supported metal catalysts on the surfaces of graphite, graphene, or carbon nanotubes, and the interactions at metal centers in metal–organic frameworks (MOFs).^9−14^ The same chemical forces active in these more complex chemical environments are also found in isolated organometallic complexes and their ions.^15−19^ Organometallic ions have been studied in mass spectrometry for many years^17−46^ and investigated extensively with theory.^47−62^ More recent experiments have applied infrared and electronic spectroscopy to these systems.^63−80^ In the present work, we investigate these interactions through electronic spectroscopy of mass-selected silver–benzene and silver–toluene cation complexes.

Metal cation−π complexes with aromatics such as benzene or toluene have been studied for many years in mass spectrometry and cluster science. Mass spectrometry studies on these complexes have employed collision-induced dissociation (CID) and related techniques to determine bond energies.^26,28,37,38,40,43,46^ Computational studies of isolated systems have also explored the bonding energetics and structures.^47−62^ Photodissociation measurements in the UV–visible region have explored the photochemistry of these systems.^20−23,32−35,39,41,45^ Infrared experiments have been reported for several metal ion–benzene complexes.^63−68^ Photoelectron spectroscopy measurements have explored the vibrational structure in cation ground states.^69−71^ Electronic spectroscopy has been studied in the UV–visible region using photodissociation for certain systems.^22,23,33−35,39,41,72^ Metal anion complexes with benzene have also been investigated.^73−76^ In recent work, our lab has developed a photofragment imaging experiment on mass-selected ions and used it to explore the bond energies of cation−π complexes.^77−80^

Perhaps the most interesting aspect of metal cation−π complexes has been the observation of photoinduced charge-transfer dissociation. In cation–molecular complexes with close ionization energies between the component species, electronic excitation can induce a charge-transfer process leading to dissociation in the excited electronic state and production of the unanticipated charged fragment having a higher ionization energy. Such processes were described initially for non-metal atmospheric ions by Bowers and co-workers.^81−83^ Our research group showed that the same kind of process occurs for metal-containing ions.^21−23,77,78,80^ Charge-transfer electronic spectra for species such as Ag^+^(benzene) were documented many years ago in solution by Mulliken and others,^84−86^ and our gas phase experiments expand on that early work. Our research group documented the charge-transfer fragmentation channel for several metal ion–benzene complexes,^21−23,77,78,80^ whereas the group of Yeh extended these studies to several other aromatic ligands.^33−35,39,41^ Kleiber and co-workers found similar photochemistry for metal ion–ethylene complexes.^87−89^ Wavelength-dependent electronic spectra were reported for some complexes,^22,23,39,41,87−89^ but these studies were limited by the tunability of available laser systems. Recent work from our lab has employed photofragment velocity-map imaging to silver–aromatic systems to investigate the energetics of the bonding in metal ion–aromatic systems.^77,78,80^ In the present report, we investigate the electronic spectroscopy of these same systems using the broad tunability of a UV–visible OPO laser system. Silver–benzene and silver–toluene ions demonstrate the characteristics of the electronic spectra of such cation−π complexes and reveal unanticipated electronic transitions.

## Methods

Ag^+^(benzene) and Ag^+^(toluene) ions were produced by laser vaporization of metal rods in a pulsed-nozzle cluster source.^90^ Benzene or toluene was introduced at their ambient vapor pressure by adding a few drops of the liquid to the gas lines. To reduce the vapor pressure, some experiments used a liquid reservoir in an ice bath. A Spectra-Physics INDI Nd:YAG laser at 355 nm was employed for the vaporization. Ions were analyzed and selected in a reflectron time-of-flight mass spectrometer. After mass selection, UV laser excitation took place in the turning region of the reflectron field, and fragment ions were analyzed by their flight times through a second field-free drift section.^91,92^ Photodissociation was accomplished with a tunable UV–visible OPO laser system (Continuum Horizon II; linewidth ∼5 cm^–1^; 1–5 mJ/pulse energy). Wavelengths were calibrated with an Avantes StarLine spectrometer. Resonance-enhanced photodissociation (REPD) spectra were recorded by measuring the yield of either the benzene or toluene cation photofragment signal as a function of the laser wavelength.

Computational studies on silver–benzene and silver–toluene complexes were carried out with the Gaussian 16 program package,^93^ using density functional theory (DFT) and the B3LYP functional with the def2-TZVP basis set. Electronic spectra were predicted using time-dependent density functional theory (TD-DFT).

## Results and Discussion

**Figure 1:**
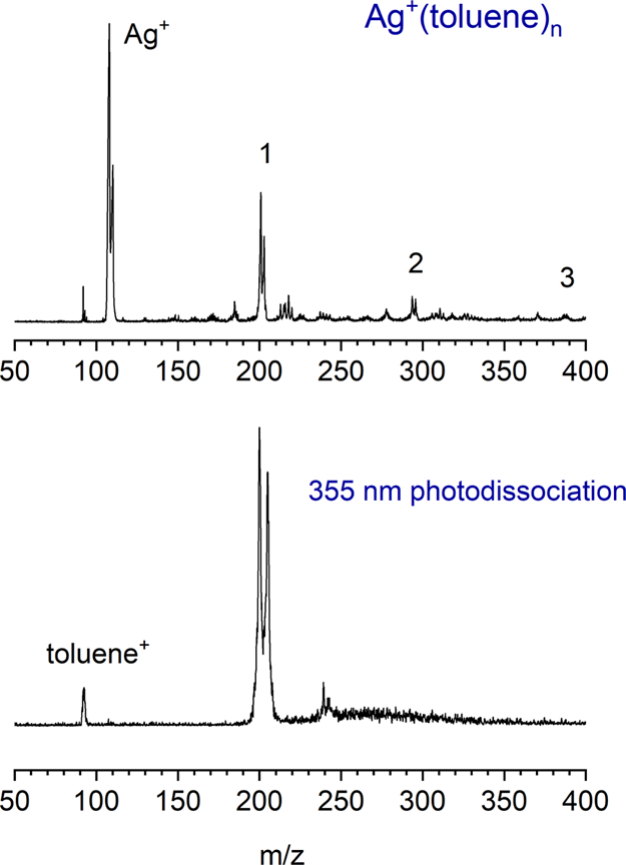
(Upper frame) Mass spectrum of the complexes produced by laser vaporization of silver in an expansion containing toluene vapor. (Bottom frame) Photodissociation mass spectrum of the mass-selected Ag^+^(toluene) ion at 355 nm, which produces the toluene cation as the only photofragment. Peaks containing silver are doubled from the two naturally occurring isotopes (107/109).

Laser vaporization produced a variety of Ag^+^(L)_*n*_ complexes for both benzene and toluene, as described in our previous work on these systems.^21−23,77,78^ The top trace of Figure 1 shows the mass spectrum for Ag^+^(toluene)_*n*_ complexes; the bottom trace shows the photodissociation mass spectrum at 355 nm (28,169 cm^–1^) in which the only fragment ion is the toluene cation. Mass peaks for ions containing silver exhibit doublets from the two naturally occurring 107/109 isotopes. Similar mass and fragmentation spectra were obtained for Ag^+^(benzene)_*n*_ complexes, with the corresponding benzene cation as the only photofragment. This fragmentation behavior was the original evidence reported many years ago by our group for the occurrence of photoinduced charge-transfer dissociation in these systems.^21−23^

**Figure 2:**
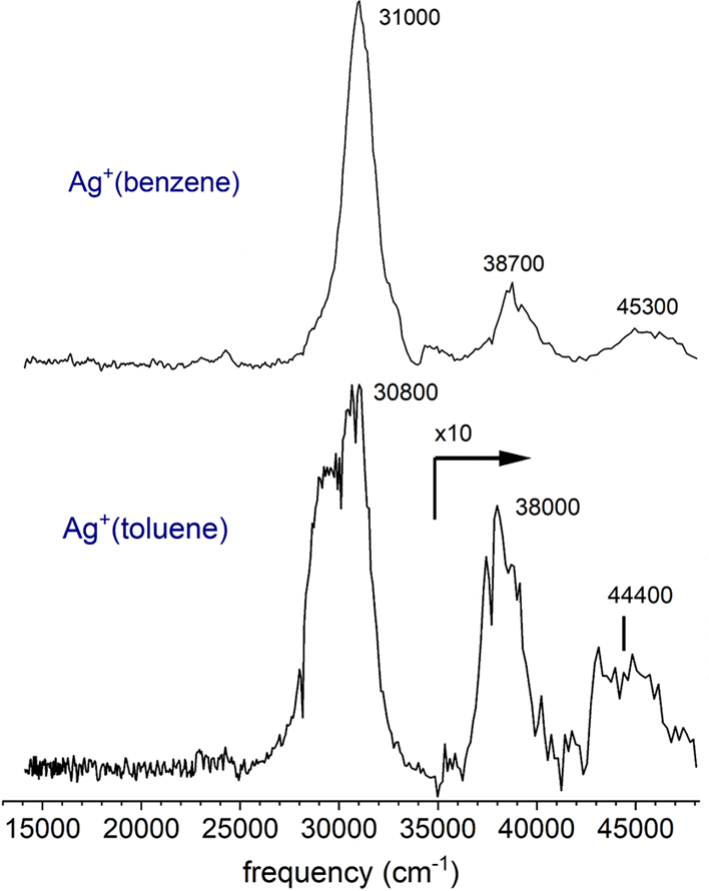
Resonance-enhanced photodissociation spectra of the Ag^+^(benzene) and Ag^+^(toluene) cations measured in the mass channels of benzene^+^ or toluene^+^ ions corresponding to photoinduced charge transfer.

In the present work, we measure the wavelength dependence of the cation fragments formed from these charge-transfer processes. Figure 2 shows a comparison of the photodissociation action spectra for Ag^+^(benzene) and Ag^+^(toluene), each measured in the mass channel of the corresponding organic cation. No other photofragments were detected throughout the region of these experiments. Both complexes have a broad, intense band in their spectra in the near-UV region near 31,000 cm^–1^ (322 nm), with two less intense features at higher energy. The toluene spectrum has a lower energy onset than the benzene spectrum, but the main features in both cases have comparable widths. The weaker bands at higher energy are at about the same positions for both complexes. The main broad feature for Ag^+^(toluene) has a reproducible dip near the middle of the band, the source of which is not clear.

**Figure 3:**
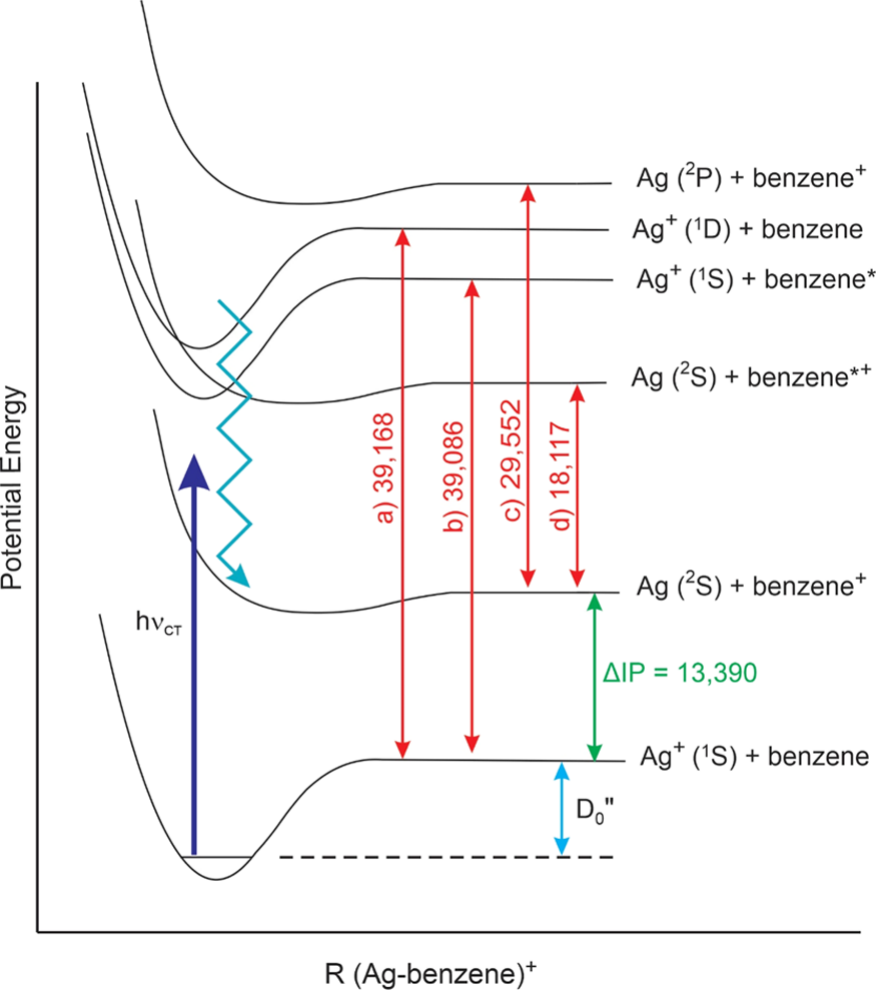
Schematic potential energy diagram for the Ag^+^(benzene) cation showing the excited states involved in the electronic spectroscopy. All units are cm^−1^.

These spectra can be interpreted with the schematic potential energy curve shown in Figure 3 for the Ag^+^(benzene) complex; similar energetics and state patterns are found for Ag^+^(toluene) as shown in Figure S1 in the Supporting Information. The ground electronic state of Ag^+^(benzene) has the charge on the silver cation because its ionization energy (7.58 eV) is lower than that of the benzene molecule (9.24 eV).^94,95^ The ground electronic state of Ag^+^(toluene) also has the charge on the silver because the ionization energy of toluene is higher (8.83 eV).^94,96^ Consistent with this, collision-induced dissociation of Ag^+^(benzene) has been studied by Armentrout and co-workers.^24^ The only fragmentation product in that experiment, in which dissociation proceeds on the ground electronic state potential energy surface, is the silver cation. The silver cation has a 4d^10^ (^1^S) electronic configuration, and its first excited state is the 4d^9^5s^1^ (^1^D) state. The ^1^S → ^1^D interval of 39,168 cm^–1^ (4.86 eV) is indicated as transition (a) in Figure 3. The first excited state of the benzene molecule is produced by the S_0_ → S_1_ transition at 39,086 cm^–1^ (4.85 eV) and is labeled as transition (b) in Figure 3.^95^ After charge transfer, the relevant species are the *neutral* silver atom in its ground 4d^10^5s^1^ (^2^S) state and the benzene *cation*. The first excited state of neutral silver is the 4d^10^5p^1^ (^2^P) state; the ^2^S → ^2^P transition occurs at 29,552 cm^–1^ (3.66 eV) and is labeled as transition (c) in the figure. The first excited state of the benzene cation lies at 18,117 cm^–1^ (2.25 eV) above the benzene cation ground state, labeled as transition (d) in Figure 3.^97,98^ Similar information is available for toluene and its cation and is presented in Figure S1 in the Supporting Information.^96−98^

As shown in Figure 3 (for benzene) and Figure S1 (for toluene), the lowest energy excited states for either the Ag^+^(benzene) or Ag^+^(toluene) complexes are the charge-transfer states correlating to the Ag (^2^S) + ligand^+^. Whereas the ground state has a charged atom interacting with a highly polarizable neutral molecule, the charge-transfer excited state has a charged molecule interacting with a neutral atom. The electrostatic interaction in the latter case is expected to be weaker, and the equilibrium bond distance is longer than in the ground state. The vertical excitation from the ground state of the Ag^+^(benzene) or Ag^+^(toluene) ions is therefore expected to carry the system to the repulsive wall of the charge-transfer excited state. Consistent with this idea, both of these complexes have been documented previously to produce significant kinetic energy release upon excitation at 355 nm (28,169 cm^–1^),^77,78^ which falls within the profile of the first band for each complex. This likely produces the main broad features detected in the experiment for Ag^+^(benzene) at 29,000–33,000 cm^–1^ and for Ag^+^(toluene) at 27,000–33,000 cm^–1^. Because the final state is repulsive with a vibrational continuum, there is no vibrational structure. However, it is not clear which of the other higher excited states of the system can account for the additional transitions seen for both Ag^+^(benzene) and Ag^+^(toluene) in the 37,000–46,000 cm^–1^ region.

To investigate the assignment of these spectra in more detail, we have conducted computational studies on the structures and thermochemistry of both of these complexes, as well as TD-DFT computations on their electronic transitions and spectra. The details of these calculations are provided in the Supporting Information. The ground state structures for both of these ions were reported in our previous work.^77,78^ The Ag^+^(benzene) complex has the silver cation located above a C–C double bond of the benzene ring rather than centered on the six-fold axis above the center of the ring. Remarkably, this asymmetric structure was suggested by Mulliken in 1952 on the basis of molecular orbital arguments.^85^ The Ag^+^(toluene) ion has the silver cation in this same kind of position above a C–C double bond of the ring, but there are two possible isomers. Isomer 1 (more stable) has the silver ion bridging 3–4 carbons away from the methyl position, and isomer 2 (relative energy, +0.83 kcal/mol) has the silver ion bridging 2–3 carbons away from this site. These structures are consistent with those obtained previously by other computational studies.^49,78^

**Figure 4:**
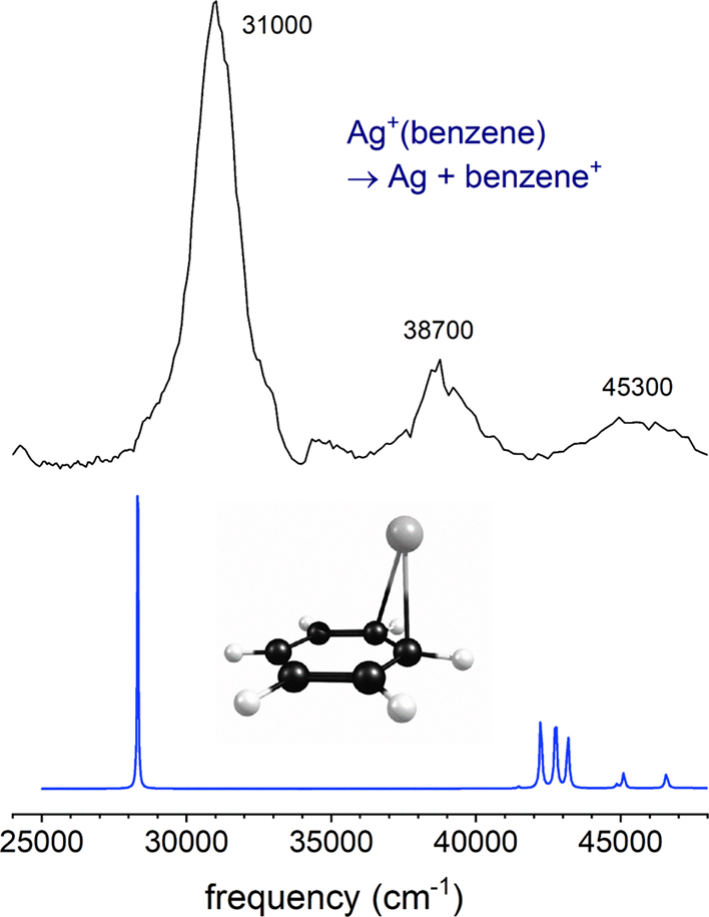
REPD spectrum of Ag^+^(benzene) compared to the spectrum predicted by time-dependent density functional theory.

Figure 4 shows the electronic spectrum measured for Ag^+^(benzene) compared to that predicted by TD-DFT (with no electronic scaling). It is well known that TD-DFT has limited quantitative reliability, and that some scaling is usually necessary to match experimental spectra.^99,100^ However, because there are no known excited states for this system, it is unclear what scaling should be used. Remarkably, the unscaled theory produces predicted transitions that match the experiment reasonably well. A strong feature corresponding to the charge-transfer electronic transition is predicted near 28,000 cm^–1^ at a position on the low-energy side of the more intense experimental band. The TD-DFT calculation does not include any vibrational structure or Franck–Condon simulation and therefore the transition predicted should correspond to the vertical transition from the *v* = 0 ground state species to the excited potential at that same structure. The predicted band is, therefore, near the position that might be expected for the band origin of the charge-transfer transition.

TD-DFT also predicts transitions at roughly the position of the second band detected in the experiment. Based on the approximate energetics, several kinds of transition are conceivable in this wavelength region. A transition at about this energy could occur for a HOMO–LUMO excitation on the benzene ligand, one with a final state corresponding to an excited state of the benzene cation, or one involving a chromophore based on silver. Of the energetically possible excitations in this region, only the formation of levels correlating to the Ag (^2^P) + benzene^+^ asymptote or that correlating to the Ag (^2^S) + benzene^+^* could lead directly to the production of the benzene cation photofragment, which is the only product detected. However, production of the Ag (^2^S) + benzene^+^* excited state would require moving one electron from the benzene π system into the silver atom ground state and also moving another electron to an excited state of the benzene cation. Such a two-electron transition is not optically allowed. There is also a problem in dissociation on the Ag (^2^P) + benzene^+^ excited state because this lies much higher than the energy of the absorption. If we assume that the ground-state dissociation energy for Ag^+^(benzene) is roughly 13,200 cm^–1^ (1.64 eV) as predicted by theory, the ΔIP value of 13,390 cm^–1^ (1.66 eV) and the Ag ^2^S → ^2^P transition energy (29,552 cm^–1^) indicate that dissociation on the Ag (^2^P) + benzene^+^ asymptote should require at least 56,000 cm^–1^. However, the higher energy transition that produces the benzene cation begins at about 37,000 cm^–1^. It is therefore unlikely that absorption involving molecular states correlating to the Ag (^2^P) + benzene^+^ asymptote can explain this transition.

**Figure 5:**
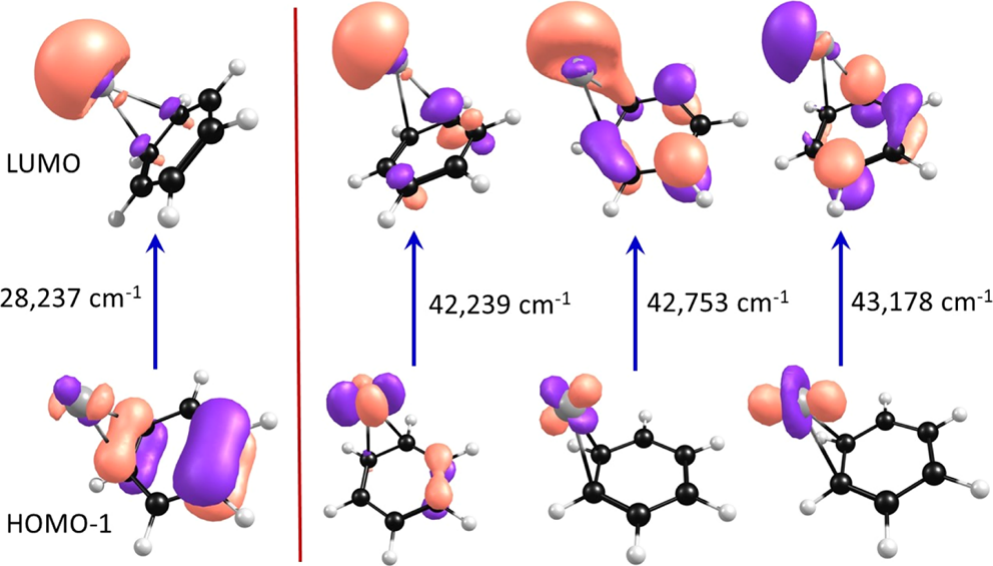
Molecular orbital charge density maps of the charge-transfer transition (left) and the metal ion-based transitions (right) of Ag^+^(benzene).

Further insight into the nature of these electronic transitions is possible from consideration of the molecular orbitals involved. According to the TD-DFT results, the main charge-transfer resonance is described by a single transition of the form HOMO −1 → LUMO, which corresponds to moving an electron from the π system of the benzene ligand to the 5s orbital on the silver. This produces a neutral silver atom in its ^2^S ground state and a charged benzene, as expected for the charge-transfer transition. The “natural transition orbitals”^101^ for this transition are shown in the left frame of Figure 5. The orange color in these maps indicates a gain in electron density during the transition, whereas the purple color indicates a loss in electron density. The shapes and colors confirm the nature of the charge-transfer transition. The π system on the benzene ligand loses electron density, and the s orbital on the silver atom gains electron density, as expected. The orbitals involved in the higher-energy transitions are more difficult to visualize, as there are 5–6 transitions contributing to each (see the Supporting Information). In this case, it is possible to plot the average of the orbitals for the group of transitions contributing to each resonance. The right side of Figure 5 shows the resulting averaged orbitals involved in the three higher energy resonances for the Ag^+^(benzene) complex. As indicated, the initial state involves taking electron density from the d orbitals on the silver atom and the final state involves placing electron density in the s orbital of the silver atom. This is exactly what happens in the Ag^+^ 4d^10^ (^1^S) → 4d^9^5s^1^ (^1^D) atomic transition. There are three transitions overlapping in the same energy region, as expected for different molecular states derived from different orientations of the Ag^+^ (^1^D) excited-state orbitals interacting with the benzene molecular ligand. Theory therefore indicates that absorption is caused by transitions on the Ag^+^(benzene) complex correlating to the Ag^+^ (^1^D) + benzene excited state. The ^1^S → ^1^D atomic transition on the isolated silver atomic ion is forbidden, but adding the benzene produces molecular states with symmetries that make allowed transitions possible. We have reported strong molecular transitions previously for Ca^+^(rare gas) and Ca^+^(acetylene) complexes correlating to a similar forbidden atomic transition.^102,103^

The other kind of electronic transition expected in the higher-energy range of this experiment is the HOMO → LUMO excitation localized on the benzene ligand. The asymptotic energy for this is labeled as transition (b) in Figure 3. According to theory, no single transition corresponds to this excitation, but rather there are several weak transitions with character mixed between silver atomic transitions and the HOMO–LUMO transition. We therefore conclude that TD-DFT is not describing the excitation properly and that the HOMO–LUMO transition provides the most likely assignment for the weak band at 45,300 cm^–1^ in the experiment.

The molecular orbital analysis explains how *absorption* happens for the higher-energy features in the spectrum, but it does not explain how the complex dissociates to produce the benzene cation, which is the only photofragment detected. It is clear from the energetics discussed earlier that dissociation cannot occur on the Ag (^2^P) + benzene^+^ potential because its asymptote lies at too high an energy. Likewise, the Ag (^2^S) + benzene^+^* potential corresponds to a two-electron transition which is also precluded. The only other potential surface leading to the benzene cation product, which is the photofragment detected, is the lower-energy Ag (^2^S) + benzene^+^ charge-transfer surface. This is the same dissociation potential leading to the benzene cation product from the main electronic transition in the 29,000–33,000 cm^–1^ region. For dissociation to occur on this potential, the Ag^+^(benzene) molecule in its excited state [either the molecular state correlating to the Ag^+^ (^1^D) + benzene asymptote or that correlating to the Ag^+^ (^1^S) + benzene*] must undergo internal conversion to the lower-energy Ag (^2^S) + benzene^+^ potential, where it then dissociates. It is also conceivable that fluorescence occurs to enable this transition. The zig-zag blue line in Figure 3 is drawn to show this transition. Either of these processes also results in a transfer of charge from the silver cation to the benzene ligand. Whereas the main electronic transition for the Ag^+^(benzene) cation in the 29,000–33,000 cm^–1^ region results from photo-induced charge transfer, this must be an example of an intramolecular excited-state charge-transfer process. Because the yield of the benzene cation approximately matches the relative intensity predicted by theory for absorption into the higher-energy states correlating to the Ag^+^ (^1^D) + benzene asymptote, the quantum yield for the intramolecular charge transfer must be close to unity. The strength of the benzene HOMO → LUMO transition is unclear because of the difficulty of TD-DFT in describing this excitation. There are many examples of charge-transfer processes between donor and acceptor complexes within the excited states of inorganic complexes, but the present system is an unusual example of this kind of process.

These assignments for the spectral features of Ag^+^(benzene) are consistent with the photofragment images of this ion at different wavelengths, which were reported previously by our lab.^78^ At 355 nm (28,169 cm^–1^), which excites on the lower-energy edge of the charge-transfer transition, most of the benzene^+^ ions formed have significant kinetic energy. This indicates that excitation at this wavelength is well above the asymptotic limit on the charge-transfer potential. The image is anisotropic, indicating prompt dissociation on the repulsive potential. Excitation at 266 nm (37,594 cm^–1^) falls in the second transition for Ag^+^(benzene). This causes significant kinetic energy release again for a sizeable fraction of the ions, but another significant fraction has near-zero kinetic energy. This suggests that a significant amount of excess energy has been lost via some process other than kinetic energy release, such as could occur if there were internal conversion or fluorescence involved in the excited-state charge-transfer process suggested above. The image at 266 nm (37,594 cm^–1^) is also much more isotropic, consistent with slower dynamics before dissociation.

**Figure 6:**
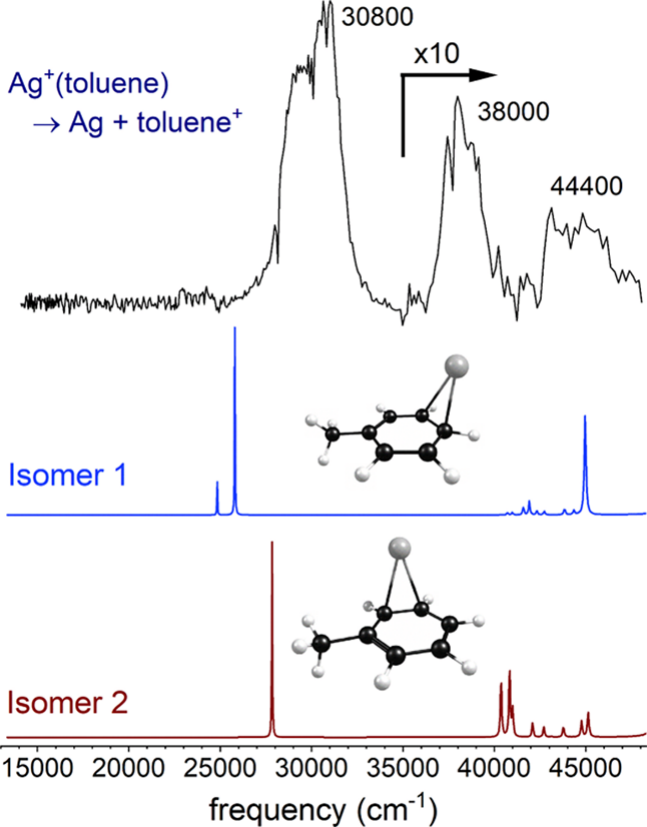
REPD spectrum of Ag^+^(toluene) compared to the spectrum predicted by time-dependent density functional theory for the two possible isomers.

The same kind of spectroscopy and dynamics are found for the Ag^+^(toluene) complex. Figure 6 shows the spectrum measured for Ag^+^(toluene) compared to that predicted by TD-DFT for the two isomers of this complex. Both isomers have a predicted band for the charge-transfer transition producing the Ag (^2^S) + toluene^+^ state, with the silver atom in its ground state. However, theory suggests that these two isomers differ in the nature of the higher energy transition, as shown in the orbital depictions in Figures S3 and S4 in the Supporting Information. Isomer 1 has a strong transition near 45,000 cm^–1^ arising from a HOMO–LUMO excitation localized on the toluene ligand, whereas isomer 2 has the same kind of transition seen for Ag^+^(benzene) correlating to the excited Ag^+^ (^1^D) + toluene state near 40,000 cm^–1^. Both of these higher-energy transitions are detected in the toluene cation mass channel; therefore, both the absorption mechanism and the intramolecular excited-state charge-transfer process must be similar to those observed for Ag^+^(benzene). The predicted positions of the two higher-energy transitions, with weak multiplet features near 40,000 and 45,000 cm^–1^, are similar for both isomers. However, the spectrum predicted for isomer 2 agrees somewhat better with the experiment, in the sense that the lower-energy transition of these two is more intense. Isomer 2 is predicted to be 0.83 kcal/mol less stable than isomer 1, but it is not clear that such a small computed energy difference is significant, given the limited reliability of DFT for the energetics of transition-metal systems.

The Supporting Information contains similar natural transition molecular orbital plots for the Ag^+^(toluene) complex, confirming the nature of the absorption processes for both electronic transitions and in turn leading to the same conclusion about intramolecular charge transfer prior to dissociation on the lower-energy charge-transfer surface. Even though DFT is recognized to have difficulties with transition-metal systems and with charge transfer^99,100^ and the molecular orbitals involved in the higher-energy transitions for both complexes are somewhat more difficult to interpret, the TD-DFT treatment seems to do a reasonable job predicting the absorption spectra for these silver cation–arene systems.

**Figure 7:**
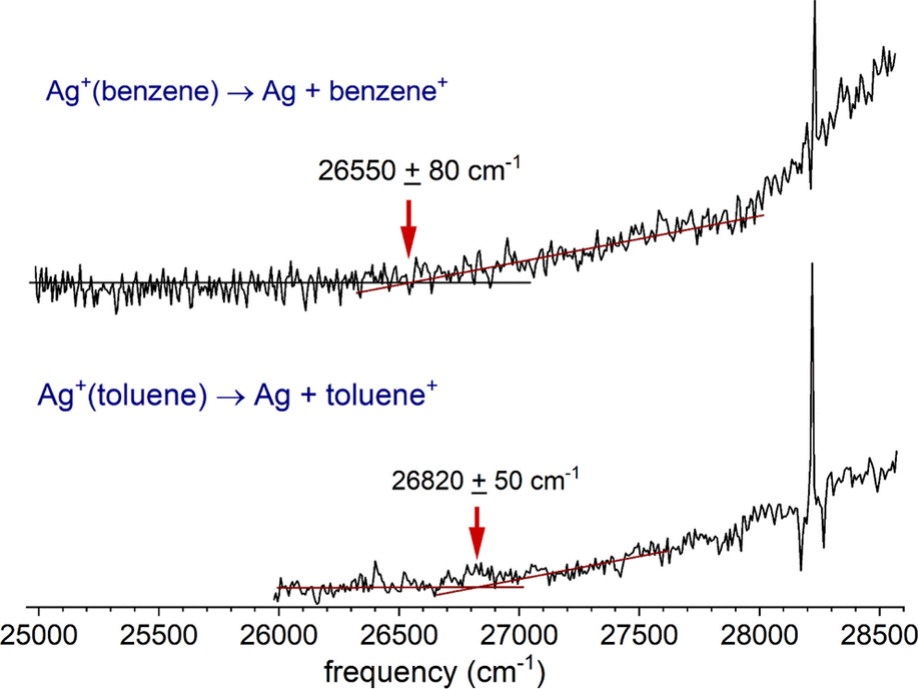
Thresholds for the onset of photodissociation signal for the Ag^+^(benzene) and Ag^+^(toluene) complexes.

The energies of the main charge-transfer transitions for both of these complexes can provide information about their ground-state dissociation energies. Referring to the potential energy curves shown in Figure 2, the relationship of the excited charge transfer Ag (^2^S) + ligand^+^ state to the ground Ag^+^ (^1^S) + ligand state can be seen. To reach the charge-transfer excited state and produce the ligand cation as the photofragment, the electronic transition must overcome the energy of the ground state well depth and the ionization potential difference between the ligand and the silver atom (*D*_0_″ + ΔIP). The photon energy that makes this possible must be greater than or equal to the asymptotic energy of the excited charge-transfer state, i.e., *E*_CT_ ≥ *D*_0_″ + ΔIP. The Franck–Condon factors for the excitation to the repulsive wall in the upper state would likely cause excitation to an energy well above the minimum threshold. Therefore, the minimum energy for the observed electronic transition provides an upper limit on the asymptotic energy. In turn, this provides an upper limit on the bond energy via the relation *D*_0_″ ≤ *E*_CT_ – ΔIP. To determine our most accurate values for the beginning of the charge-transfer signal, we have scanned the threshold regions of the spectra for Ag^+^(benzene) and Ag^+^(toluene) at higher resolution (0.1 nm step size) with more signal averaging. These threshold spectra are shown in Figure 7. The thresholds for Ag^+^(benzene) and Ag^+^(toluene) are, respectively, 26,550 and 26,820 ± 100 cm^–1^ (3.29 and 3.33 ± 0.012 eV). The error bars in these limits are relatively small because of the laser linewidth and the relatively sharp onset of signal in the experiment. In our previous work, we reported the threshold for charge-transfer signal in Ag^+^(benzene) at 418 nm (23,920 cm^–1^) and that for Ag^+^(toluene) at 385 nm (25,970 cm^–1^).^22,23^ Those values are lower than the ones reported here because the ions in those experiments were expanded in helium instead of argon and were likely hot internally. We conducted systematic studies on this effect in our imaging experiments and determined that argon expansions were essential to obtain cold ions.^77,78^ It therefore makes sense that the current thresholds are higher in energy than those reported previously and are also more reliable.

Using these new threshold energies and the ionization potentials for silver, benzene and toluene (7.58, 9.24, and 8.83 eV, respectively), the upper limits on the Ag^+^(benzene) and Ag^+^(toluene) dissociation energies are 1.63 and 2.08 ± 0.012 eV (37.6 and 48.0 ± 0.3 kcal/mol), respectively. These limits on the dissociation energies in these complexes can be compared to similar upper limits obtained recently from photofragment imaging experiments.^78^ The imaging values of *D*_0_″ ≤ 28.9 and 35.9 ± 3.2 kcal/mol for Ag^+^(benzene) and Ag^+^(toluene), respectively, are both lower than the values derived here from the spectroscopic thresholds. This is completely understandable because of the effects of the Franck–Condon factors in these optical transitions, as noted above. It is likely that the thresholds detected here are well above the asymptotic limits for the charge-transfer excited states. The photofragment imaging experiment detects kinetic energy release in the fragments as they are ejected off the repulsive wall of the charge-transfer excited states and is not as sensitive to this same kind of Franck–Condon effect. In the scanned spectroscopy experiment, no signal is detected until an energy when the Franck–Condon factors allow it. But even at the threshold for F–C allowed absorption, the ions can be ejected with kinetic energy, extending the threshold determination downward in energy. The imaging experiments are therefore likely to provide tighter upper limits on these dissociation energies. However, the resolution in the kinetic energy determination is worse than that in the spectroscopic threshold, leading to larger error bars.

The dissociation energy limits determined here can also be compared to the dissociation energy determined by Chen and Armentrout for Ag^+^(benzene) using collision-induced dissociation (35.4 ± 2.3 kcal/mol)^25^ and to the upper limits for Ag^+^(benzene) and Ag^+^(toluene) determined with photodissociation thresholds by Afzaal and Freiser (55 ± 5 and 60 ± 5 kcal/mol).^26^ As discussed in our paper on imaging, our dissociation upper limits derived from imaging are systematically lower than the CID values but almost overlapping if the error bars in both experiments are considered. Our value here for the upper limit of the Ag^+^(benzene) dissociation energy from the spectroscopic threshold lies just above the Armentrout CID value.^25^ Our values and those of Armentrout are far below the values obtained by Afzaal and Freiser.^26^ Afzaal and Freiser used longer excitation periods for many laser shots, and their experiment seems to have had very poor signal levels. Our computational values for the Ag^+^(benzene) and Ag^+^(toluene) dissociation energies are 37.8 and 40.0/40.8 (isomer 1/2) at the DFT/B3LYP/def2-TZVP level. These are consistent with the spectroscopic threshold derived here but significantly higher than the values obtained from photofragment imaging. As noted in our previous work, these computed values were investigated with several different functionals without finding any significant variations.^78^ However, our experience from studies on many different transition-metal complexes is that dissociation energies derived from DFT computations are systematically high. Because of the several dissociation energy values now available for these silver–arene ions from different experiments, these systems may provide benchmarks for studies at higher levels of theory. Investigations of the effects of spin–orbit interaction and relativity would also be highly desirable.

**Figure 8:**
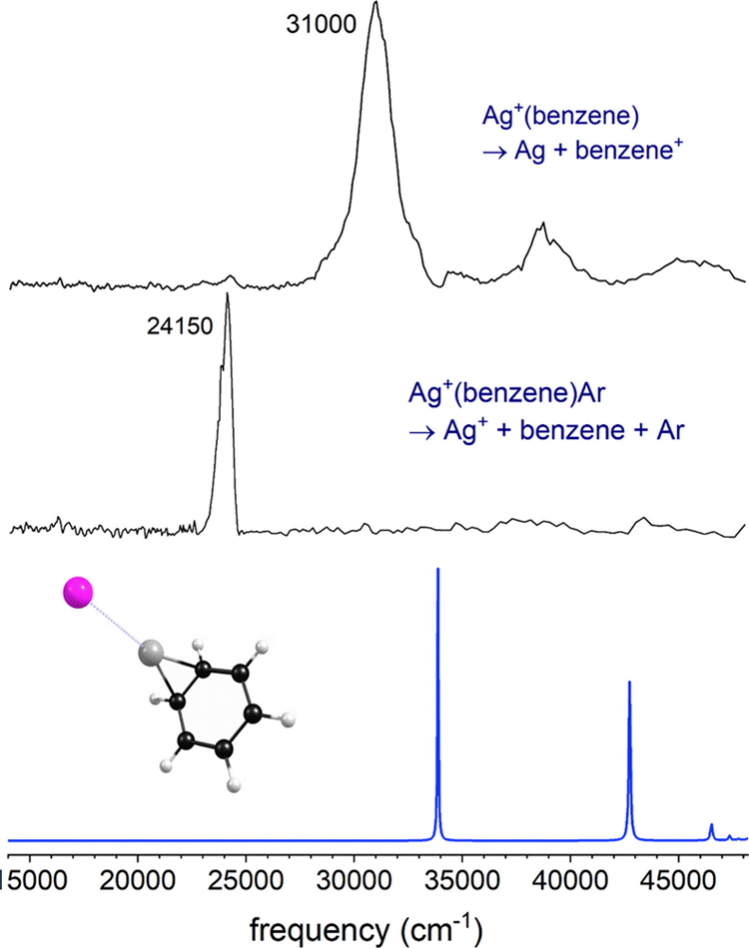
Photodissociation spectrum of Ag^+^(benzene) compared to that of Ar–Ag^+^(benzene). The photofragment from Ag^+^(benzene) is the benzene^+^ cation, whereas that from Ar–Ag^+^(benzene) is Ag^+^.

**Figure 9:**
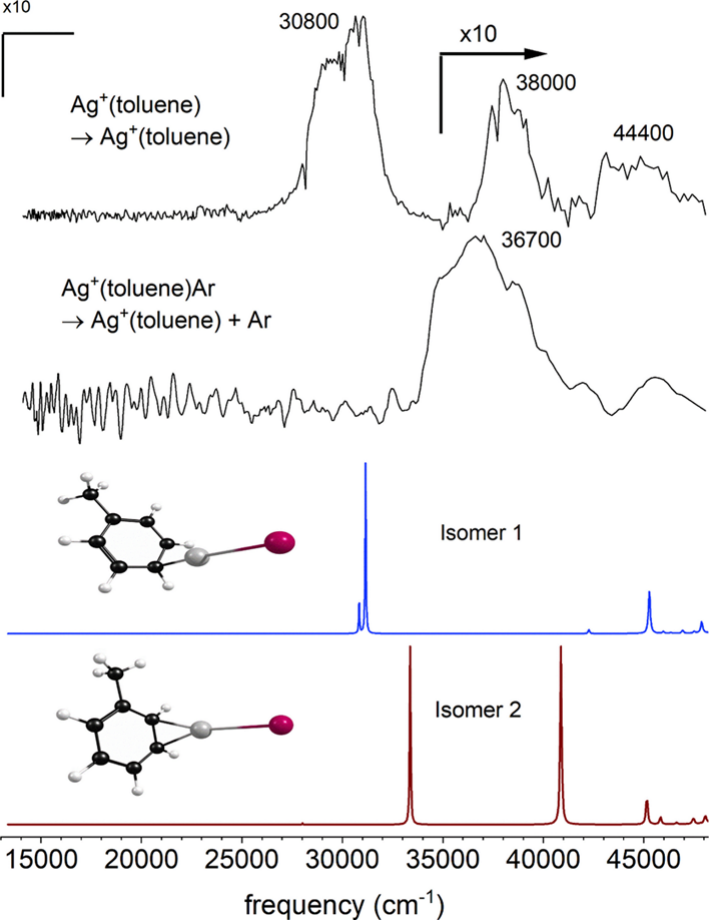
Photodissociation spectrum of Ag^+^(toluene) compared to that for Ar–Ag^+^(toluene). The photofragment from Ag^+^(toluene) is the toluene^+^ cation, whereas that from Ar–Ag^+^(toluene) is Ag^+^(toluene).

As an additional consideration, we decided to measure these same UV spectra using rare gas tagging with argon. Because we used argon as the expansion gas in these experiments, the tagged ions were produced along with the tag-free ions without any change in instrumental conditions. Tagging is employed with infrared spectroscopy by many labs and is usually believed to exhibit minimum perturbations on spectra while enhancing photodissociation yields. Some researchers have also employed it for UV–visible spectroscopy of ions. However, there are few examples of such electronic spectra measured with and without tagging with which to evaluate its actual effect on these spectra. The spectra for Ag^+^(benzene)Ar and Ag^+^(toluene)Ar are presented in Figures 8 and 9, where they are compared to the tag-free spectra and to the predictions of TD-DFT. The signal levels in the tagged spectra are worse than those in the tag-free spectra because the tagged ion signals were an order of magnitude smaller than those of the tag-free ions. Theory finds that the argon tag atom for both complexes is bound to the silver cation opposite the benzene or toluene ligand.

As shown in these two figures, argon tagging has a significant and surprising effect on these spectra. In both cases, the spectra measured with tagging are shifted dramatically from those measured for the tag-free ions. However, the direction of the shift is opposite, with an almost 7000 cm^–1^ shift to lower energy for the Ar–Ag^+^(benzene) spectrum and a shift of almost 6000 cm^–1^ to higher energy for the Ar–Ag^+^(toluene) spectrum. TD-DFT predicts a shift to higher energy for both complexes. This is consistent with a stronger solvent effect on the ground state, which has the charge localized on the silver atom, than in the excited state, which has the charge delocalized on the benzene. The resulting shift of the spectrum to higher energy is reproduced for the Ar–Ag^+^(toluene) complex. However, the Ar–Ag^+^(benzene) complex spectrum is strongly red-shifted by the tagging, which is inconsistent with the predictions of theory. Its spectrum is also much narrower. Moreover, the tagging causes unexpected trends in the fragmentation behavior. In the case of Ar–Ag^+^(toluene), the fragment ion detected is the Ag^+^(toluene) ion, produced by the elimination of argon. Even though the absorption is still into the charge-transfer resonance, there is no production of the toluene cation. In the case of the Ar–Ag^+^(benzene) ion, the fragment detected is the Ag^+^ ion, produced by the loss of both argon and neutral benzene. Apparently, the charge-transfer electronic excitation causes the absorption in these systems, but the presence of the argon significantly perturbs the fragmentation process. It is understandable that a charge-transfer electronic transition might be sensitive to a solvation interaction, but it is not clear how to explain the change in dissociation behavior or the difference in the spectral trends for the benzene versus toluene complexes. In any event, argon tagging in these systems is most certainly not inert.

## Conclusions

New electronic spectroscopy measurements are reported here for the Ag^+^(benzene) and Ag^+^(toluene) ion–molecule complexes in the UV region of the spectrum. Both of these ions exhibit a broad electronic resonance in the near-UV corresponding to charge-transfer photodissociation, producing either the benzene cation or the toluene cation respectively. The dissociation occurs on the Ag (^2^S) + ligand^+^ excited-state potential. The full wavelength dependence of the charge-transfer process is reported for the first time, showing the broad signal resulting from excitation to the repulsive wall of the charge-transfer excited-state potential. New electronic transitions are detected for both complexes at higher energies correlating to the Ag (^1^D) + ligand asymptote and HOMO → LUMO excitation on the benzene ligand. Following each of these kinds of excitations, intramolecular charge transfer occurs, leading to dissociation on the lower-energy Ag (^2^S) + ligand^+^ potential. Energetic cycles using the thresholds for the main charge-transfer processes for both ions provide upper limits on the respective cation–arene bond energies. However, the limits obtained are much higher than those derived from recent photofragment imaging experiments on these same ions. The differences between the two experiments are understandable and are attributed to the higher-energy spectroscopic thresholds caused by the Franck–Condon intensities of the charge-transfer transitions. Argon tagging produces unanticipated and unexplained shifts in these charge-transfer spectra requiring additional study. Silver cation–arene complexes exhibit fascinating electronic spectroscopy and photodissociation dynamics and may provide benchmark systems for more advanced computational studies.

## Supporting Information Available

Full citation for ref (93) and details of DFT computations performed, including the structures and energetics for each of the cluster ions considered (PDF)
