# Supplementary material for: Charge-Transfer Spectroscopy of Ag+(Benzene) and Ag+(Toluene)
Source: J Phys Chem A. 2023 May 25;127(22):4822–31. doi: 10.1021/acs.jpca.3c01790 (PMC10258802; doi:10.1021/acs.jpca.3c01790)
Supplement: Supplementary file 1 — jp3c01790_si_001.pdf [file jp3c01790_si_001.pdf]

Supporting Information:

Charge-Transfer Spectroscopy of Ag^+(Benzene) and Ag^+(Toluene)

Jason E. Colley, Dylan S. Orr, Michael A. Duncan*

Department of Chemistry, University of Georgia, Athens, Georgia 30602, U.S.A.

*Email: maduncan@uga.edu

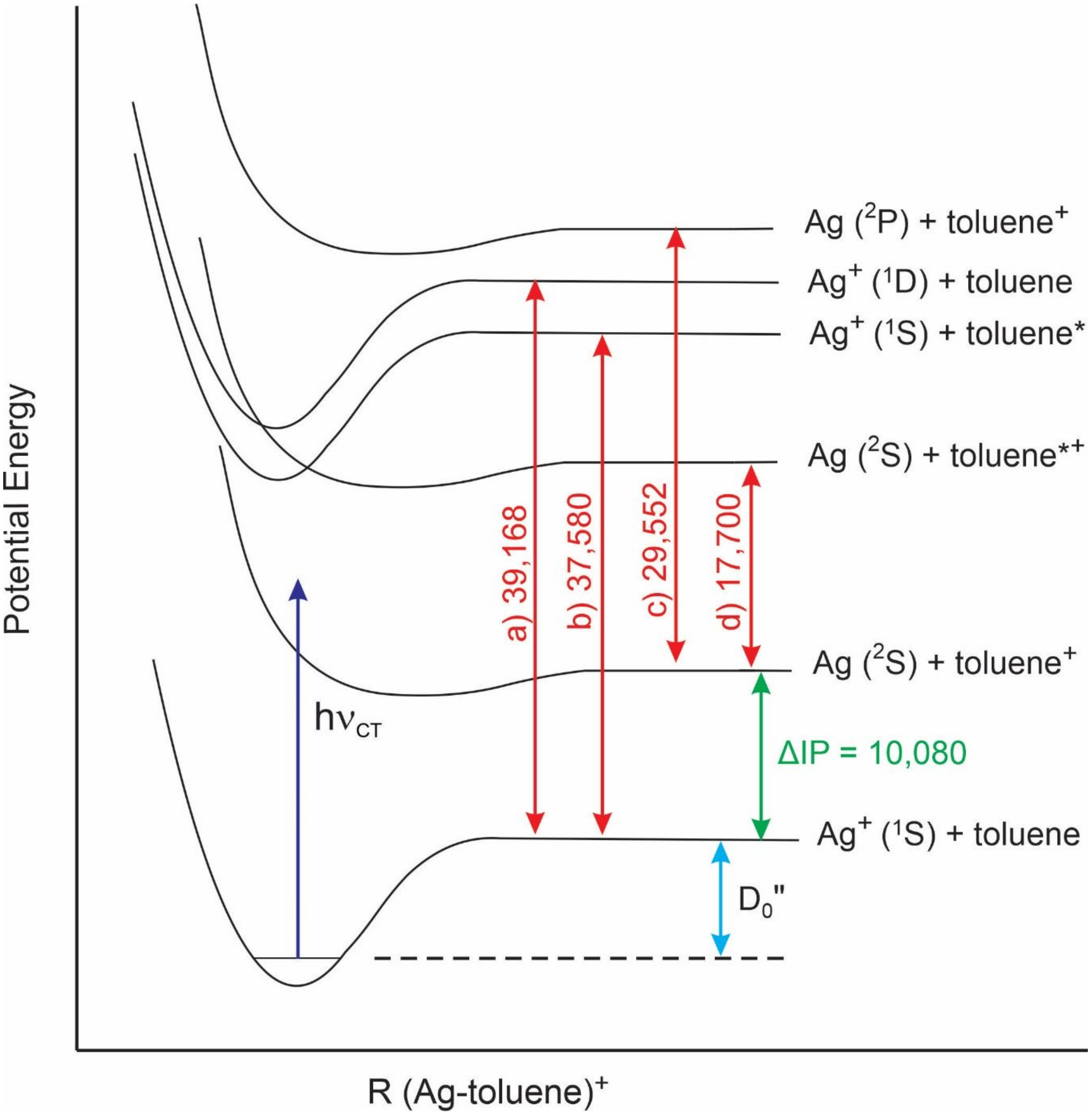

Figure S1. The schematic potential energy curves for the Ag^+(toluene) cation. Transition a) is the Ag^+ $^1S \rightarrow {}^1D$ interval; transition b) is the $S_0 \rightarrow S_1$ HOMO-LUMO transition of toluene; transition c) is the Ag $^2S \rightarrow {}^2P$ interval; transition d) is the ground $\rightarrow$ excited state interval of the toluene cation.

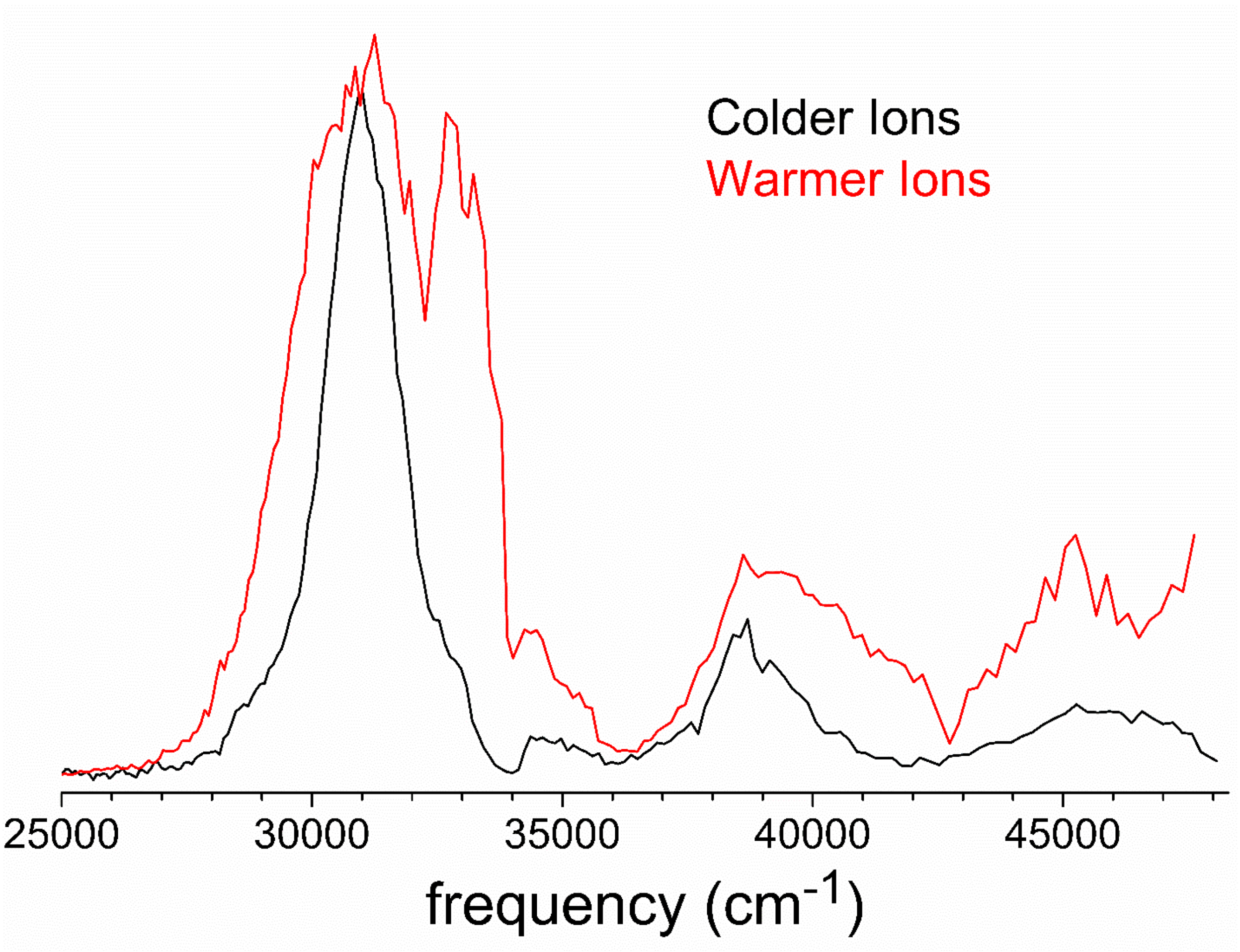

Figure S2. A comparison of the Ag^{+}(benzene) charge transfer photodissociation spectra with warmer and colder ion conditions. The warmer conditions employed a slightly different vaporization laser power and timing with respect to the gas pulse.

Full citation for reference 93:

Frisch, M. J.; Trucks, G. W.; Schlegel, H. B.; Scuseria, G. E.; Robb, M. A.; Cheeseman, J. R.; Scalmani, G.; Barone, V.; Peterson, G. A.; Nakatsuji, H.; Li, X.; Caricato, M.; Marenich, A. V.; Bloino, J.; Janesko, B. G.; Gomperts, R.; Mennucci, B.; Hratchian, H. P.; Ortiz, J. V.; Izmaylov, A. F.; Sonnenberg, J. L.; Williams-Young, D.; Ding, F.; Lipparini, F.; Egidi, F.; Goings, J.; Peng, B.; Petrone, A.; Henderson, T.; Ranasinghe, D.; Zakrzewski, V. G.; Gao, J.; Rega, N.; Zheng, G.; Liang, W.; Hada, M.; Ehara, M.; Toyota, K.; Fukuda, R.; Hasegawa, J.; Ishida, M.; Nakajima, T.; Honda, Y.; Kitao, O.; Nakai, H.; Vreven, T.; Throssell, K.; Montgomery, Jr, J. A.; Peralta, E.; Ogliaro, F.; Bearpark, M. J.; Heyd, J. J.; Brothers, E. N.; Kudin, K. N.; Staroverov, V. N.; Keith, T. A.; Kobayashi, R.; Normand, J.; Raghavachari, K.; Rendell, A. P.; Burant, J. C.; Iyengar, S. S.; Tomasi, J.; Cossi, M.; Millam, J. M.; Klene, M.; Adamo, C.; Cammi, R.; Ochterski, J. W.; Martin, R. L.; Morokuma, K.; Farkas, O.; Foresman, J. B.; Fox, D. J. Gaussian 16 (Revision C.01) Gaussian, Inc., Wallingford CT, 2019.

All calculations were carried out using an “ultrafine” integration grid, and the optimization threshold for energy and structure optimizations were set to “tight.” The “stable=opt” keyword was used on all structures to check for electronic wavefunction stability. All electronic energies are ZPVE corrected.

Ag^+(benzene)

B3LYP/def2-TZVP

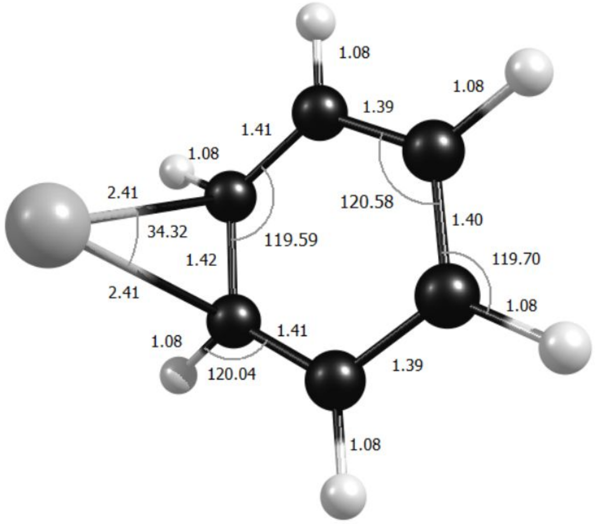

Coordinates:

Ag	-3.954172000	-4.009443000	1.549346000
C	-5.310836000	-2.025712000	1.388959000
C	-6.484749000	-2.325056000	2.100660000
C	-4.131566000	-1.667682000	2.098497000
H	-7.389206000	-2.573120000	1.561184000
H	-3.266517000	-1.302015000	1.556603000
C	-6.491832000	-2.242004000	3.485426000
C	-4.154875000	-1.617417000	3.502257000
H	-7.406029000	-2.438971000	4.029813000
H	-3.265392000	-1.320782000	4.042099000
C	-5.332764000	-1.889815000	4.182687000
H	-5.357820000	-1.816535000	5.262000000
H	-5.340231000	-1.930338000	0.309247000

Ag$^+$(benzene)
B3LYP/def2-QZVP

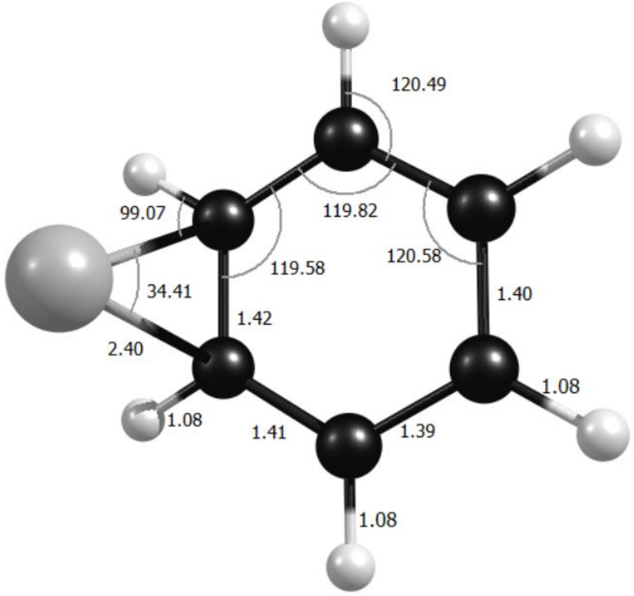

Coordinates:

Ag	-3.956477000	-4.003843000	1.555160000
C	-5.310730000	-2.026123000	1.388813000
C	-6.484372000	-2.324581000	2.100549000
C	-4.131697000	-1.668447000	2.098038000
H	-7.387172000	-2.573523000	1.561811000
H	-3.268228000	-1.301762000	1.557284000
C	-6.491254000	-2.242018000	3.484749000
C	-4.155203000	-1.617868000	3.501504000
H	-7.404004000	-2.439181000	4.028418000
H	-3.266384000	-1.323241000	4.040438000
C	-5.332456000	-1.890344000	4.181747000
H	-5.357164000	-1.817962000	5.259574000
H	-5.340845000	-1.929997000	0.310693000

Ag$^+$(benzene)
M06L/def2-TZVP

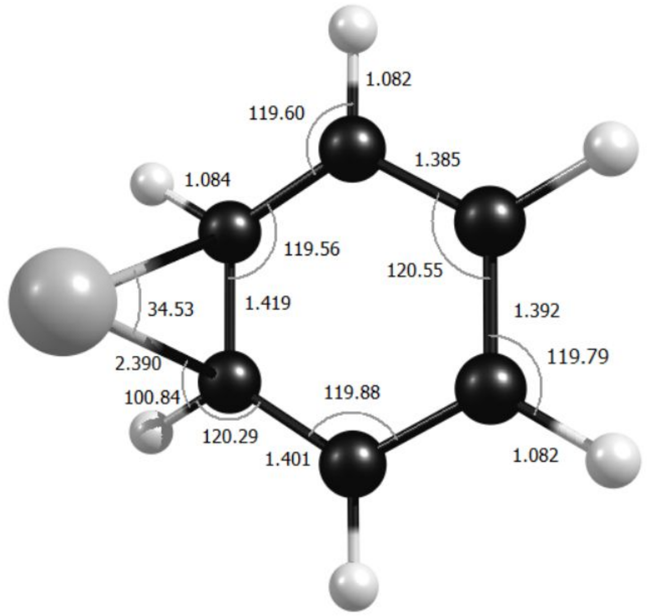

Coordinates:

Ag	-4.013605000	-3.992025000	1.651544000
C	-5.312584000	-2.003099000	1.382993000
C	-6.477375000	-2.319224000	2.093600000
C	-4.136110000	-1.646410000	2.090831000
H	-7.380827000	-2.570408000	1.554117000
H	-3.267627000	-1.291656000	1.547674000
C	-6.480458000	-2.255173000	3.477545000
C	-4.154708000	-1.615805000	3.491024000
H	-7.391183000	-2.467811000	4.021298000
H	-3.263935000	-1.323706000	4.031023000
C	-5.325712000	-1.905660000	4.172260000
H	-5.344882000	-1.848609000	5.252389000
H	-5.336982000	-1.919302000	0.302477000

Ag^+(benzene)
M06L/def2-QZVP

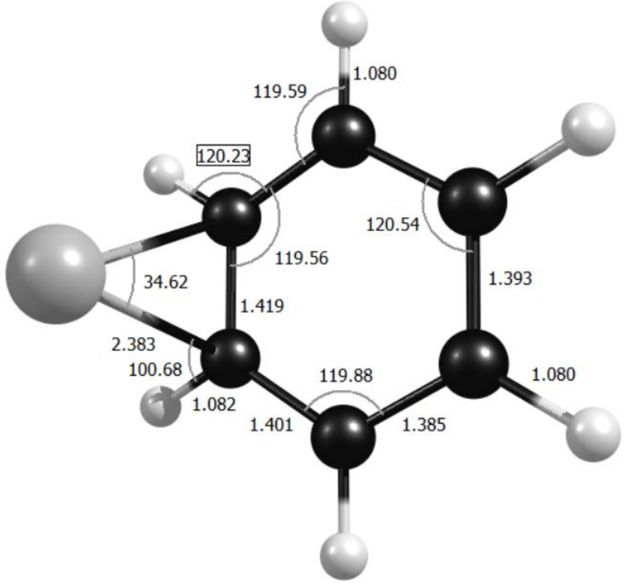

Coordinates:

Ag	-4.010512000	-3.985852000	1.643729000
C	-5.312441000	-2.005435000	1.383505000
C	-6.478082000	-2.319263000	2.094267000
C	-4.135829000	-1.648696000	2.091195000
H	-7.379026000	-2.571917000	1.555906000
H	-3.269024000	-1.294137000	1.549800000
C	-6.481455000	-2.253732000	3.478232000
C	-4.154838000	-1.615640000	3.491606000
H	-7.390193000	-2.465581000	4.021095000
H	-3.265211000	-1.326015000	4.030168000
C	-5.326178000	-1.904050000	4.173064000
H	-5.345403000	-1.846822000	5.250902000
H	-5.337797000	-1.921749000	0.305308000

Ag^{+}(toluene) Isomer 1
B3LYP/def2-TZVP

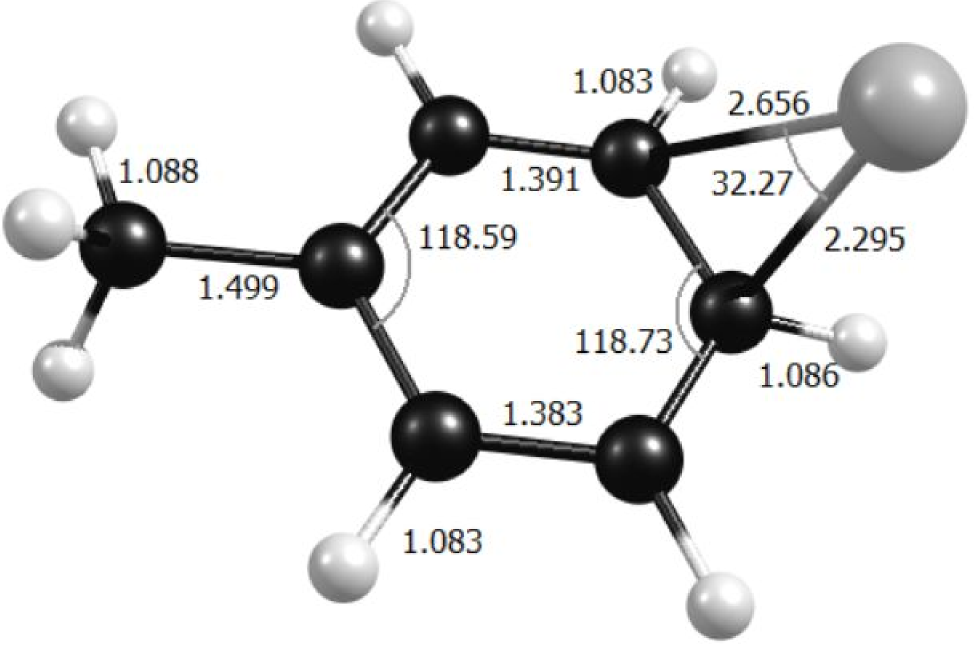

Coordinates:

Ag	1.849844000	-0.005748000	-0.422098000
H	-1.431677000	-1.320960000	1.760431000
C	-3.191267000	-0.936785000	-0.290228000
H	-3.409123000	-1.681287000	0.473052000
H	-3.088304000	-1.451307000	-1.249213000
H	-4.051932000	-0.270846000	-0.378723000
C	-1.949793000	-0.158827000	0.028597000
C	-1.571232000	0.929970000	-0.775055000
C	-0.447746000	1.683513000	-0.486627000
C	0.348083000	1.365851000	0.641677000
C	-0.020501000	0.256174000	1.445744000
C	-1.149552000	-0.490553000	1.125930000
H	-2.186742000	1.200252000	-1.624239000
H	-0.205202000	2.553107000	-1.083851000
H	1.081674000	2.079039000	1.005222000
H	0.531208000	0.045019000	2.353460000

Ag$^+$(toluene) Isomer 2
B3LYP/def2-TZVP

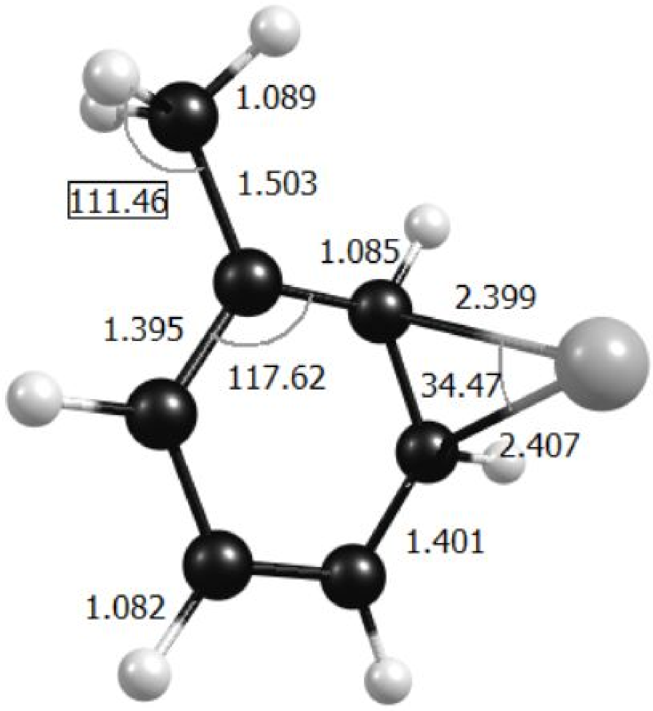

Coordinates:

Ag	1.472048000	-0.695612000	0.040941000
H	-0.596032000	-1.000617000	1.879445000
C	-2.498000000	-1.570334000	-0.009275000
H	-2.068453000	-2.432933000	0.499013000
H	-2.701130000	-1.842249000	-1.045126000
H	-3.460825000	-1.357605000	0.463017000
C	-1.606505000	-0.363982000	0.077777000
C	-1.763255000	0.717394000	-0.789871000
C	-0.990158000	1.871898000	-0.672703000
C	-0.026583000	1.986209000	0.320613000
C	0.149819000	0.930939000	1.225024000
C	-0.635821000	-0.250185000	1.096656000
H	-2.517897000	0.664399000	-1.565125000
H	-1.153441000	2.693418000	-1.358068000
H	0.552633000	2.893340000	0.427718000
H	0.783281000	1.059083000	2.095787000

Ag$^+$(toluene) Isomer 1
B3LYP/def2-QZVP

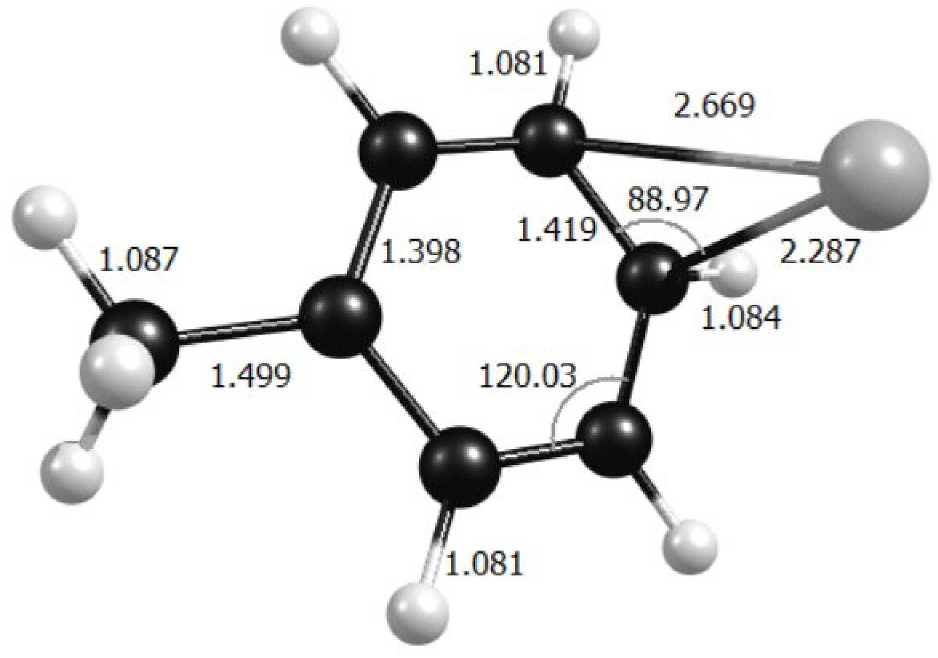

Coordinates:

Ag	1.833108000	0.026036000	-0.473714000
H	-1.425708000	-1.332407000	1.749351000
C	-3.190735000	-0.935525000	-0.289765000
H	-3.483020000	-1.584480000	0.531675000
H	-3.028833000	-1.561340000	-1.170153000
H	-4.022178000	-0.270969000	-0.521200000
C	-1.952214000	-0.154485000	0.032430000
C	-1.577967000	0.940549000	-0.762521000
C	-0.452119000	1.690173000	-0.472768000
C	0.351116000	1.359421000	0.646490000
C	-0.014050000	0.242369000	1.441072000
C	-1.145867000	-0.497770000	1.121957000
H	-2.197980000	1.220374000	-1.603215000
H	-0.213929000	2.565817000	-1.060123000
H	1.083532000	2.069219000	1.014715000
H	0.544582000	0.019628000	2.339846000

Ag$^+$(toluene) Isomer 2
B3LYP/def2-QZVP

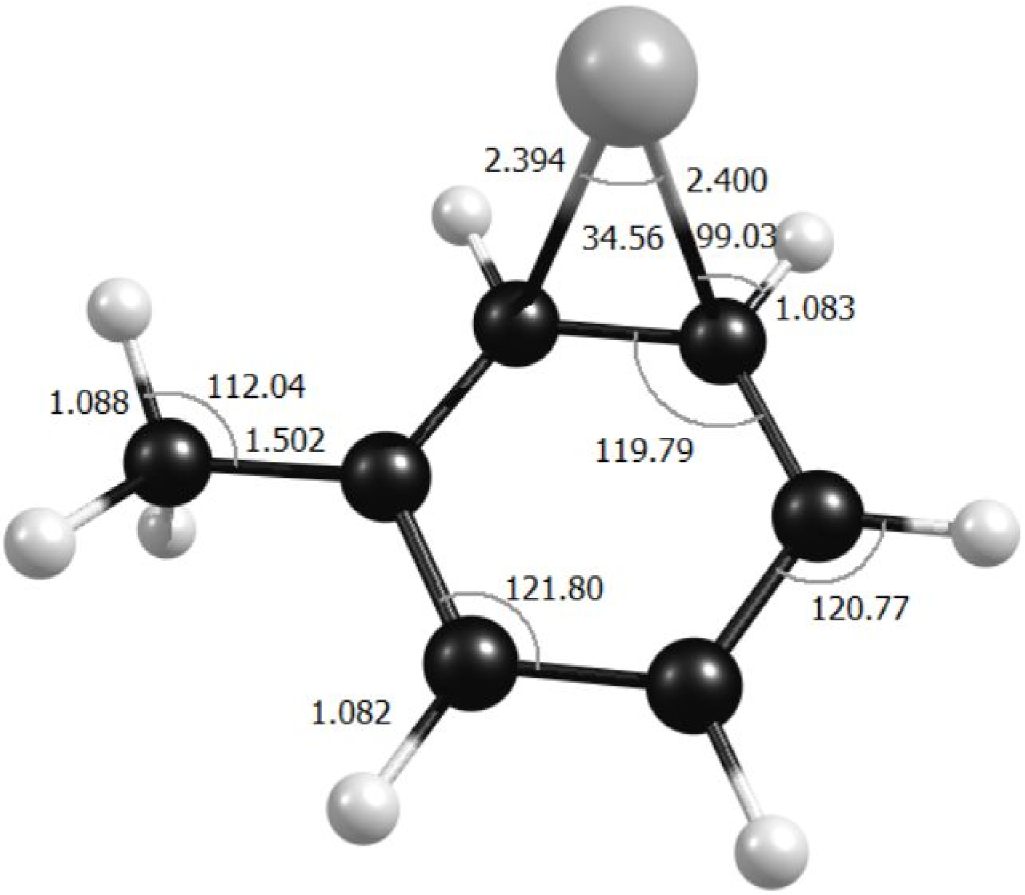

Coordinates:

Ag	1.467355000	-0.688632000	0.044993000
H	-0.599926000	-0.998373000	1.880074000
C	-2.497870000	-1.569843000	-0.009087000
H	-2.059769000	-2.437330000	0.479877000
H	-2.718820000	-1.828448000	-1.042864000
H	-3.450827000	-1.364526000	0.482432000
C	-1.606127000	-0.363750000	0.077472000
C	-1.760660000	0.715762000	-0.791816000
C	-0.987728000	1.869871000	-0.674516000
C	-0.026929000	1.985239000	0.320462000
C	0.148071000	0.931040000	1.226059000
C	-0.637220000	-0.249737000	1.097493000
H	-2.511953000	0.661499000	-1.568039000
H	-1.148547000	2.689119000	-1.360744000

H	0.552585000	2.890436000	0.426764000
H	0.778049000	1.060835000	2.097262000

Ag^{+}(toluene) Isomer 1
M06L/def2-TZVP

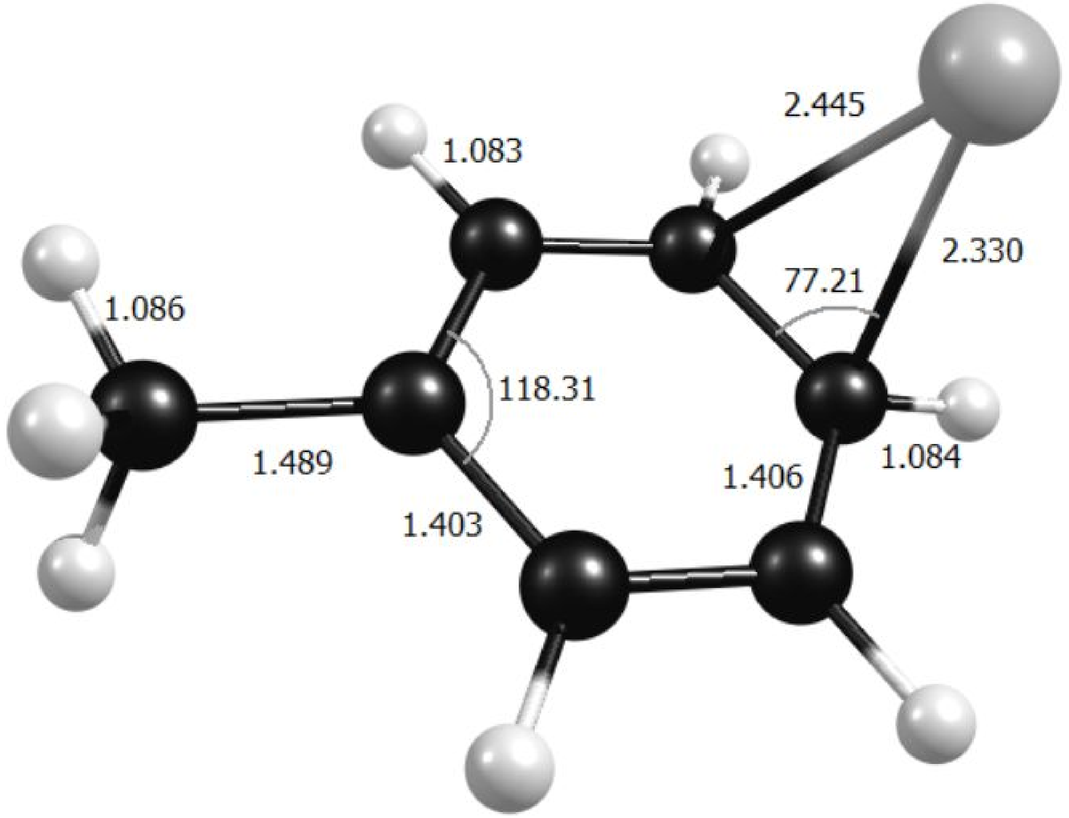

Coordinates:

Ag	1.691129000	-0.267964000	-0.230257000
H	-1.386949000	-1.313267000	1.738533000
C	-3.156487000	-0.944591000	-0.296670000
H	-3.271626000	-1.801418000	0.361066000
H	-3.144593000	-1.300575000	-1.326655000
H	-4.046906000	-0.322876000	-0.197286000
C	-1.931977000	-0.157133000	0.016605000
C	-1.574750000	0.942096000	-0.778160000
C	-0.465759000	1.713679000	-0.495116000
C	0.329771000	1.418697000	0.625505000
C	-0.011341000	0.301705000	1.429702000
C	-1.124610000	-0.474468000	1.106108000
H	-2.196404000	1.199845000	-1.627004000
H	-0.229522000	2.575408000	-1.105427000
H	1.096581000	2.111433000	0.953676000
H	0.531182000	0.116042000	2.349457000

Ag^+(toluene) Isomer 2
M06L/def2-TZVP

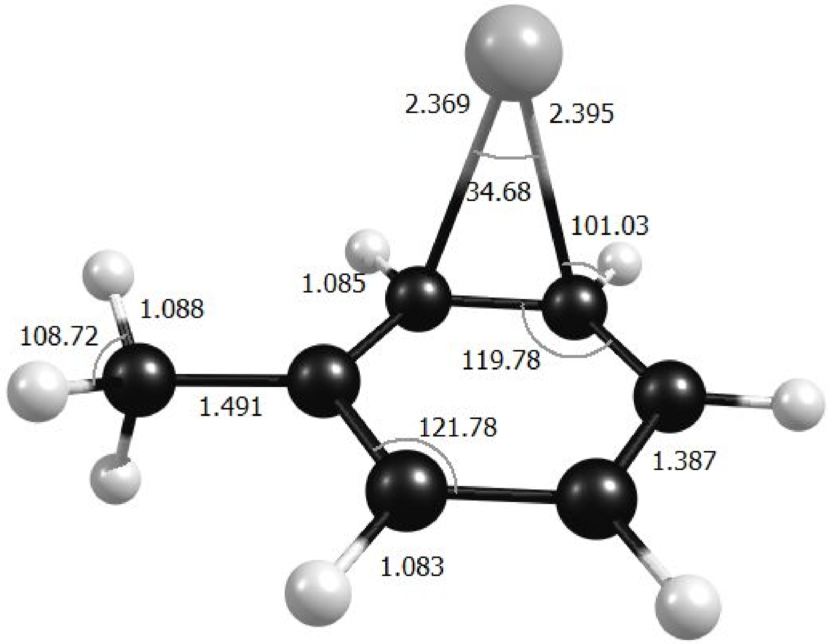

Coordinates:

Ag	-1.038505000	-1.241570000	0.425747000
H	0.034473000	0.694357000	2.119505000
C	-0.107527000	2.605781000	0.176964000
H	-0.925735000	2.618655000	0.893422000
H	-0.487967000	2.918276000	-0.793052000
H	0.606401000	3.367903000	0.494282000
C	0.561598000	1.275302000	0.106928000
C	1.323785000	0.902629000	-0.999311000
C	2.005639000	-0.306449000	-1.044022000
C	1.948150000	-1.194796000	0.019259000
C	1.209648000	-0.853397000	1.154528000
C	0.507654000	0.380620000	1.194528000
H	1.395393000	1.584484000	-1.837862000
H	2.596926000	-0.553257000	-1.915888000
H	2.497683000	-2.125754000	-0.007640000
H	1.249589000	-1.473249000	2.042980000

Ag^{+}(toluene) Isomer 1
M06L/def2-QZVP

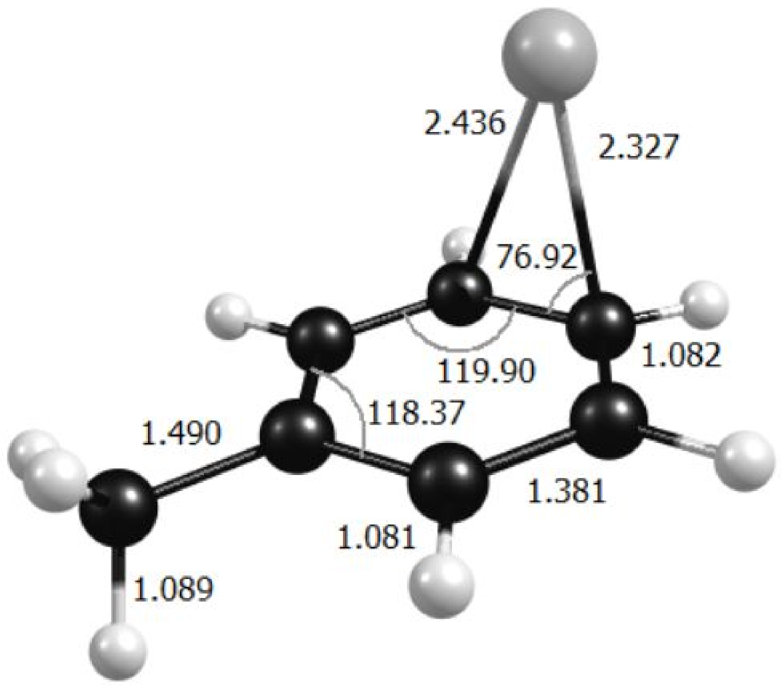

Coordinates:

Ag	1.690774000	-0.269094000	-0.223707000
H	-1.382430000	-1.315543000	1.733734000
C	-3.157225000	-0.944808000	-0.296846000
H	-3.282246000	-1.788409000	0.373094000
H	-3.131353000	-1.318246000	-1.318650000
H	-4.044519000	-0.318788000	-0.220737000
C	-1.932674000	-0.155824000	0.017966000
C	-1.578726000	0.945878000	-0.775176000
C	-0.468699000	1.716702000	-0.493226000
C	0.330724000	1.418007000	0.623801000
C	-0.007952000	0.299149000	1.426533000
C	-1.123291000	-0.476095000	1.104688000
H	-2.201432000	1.204716000	-1.620066000
H	-0.233225000	2.577053000	-1.101711000
H	1.095989000	2.109468000	0.950635000
H	0.534025000	0.112447000	2.343748000

Ag^+(toluene) Isomer 2
M06L/def2-QZVP

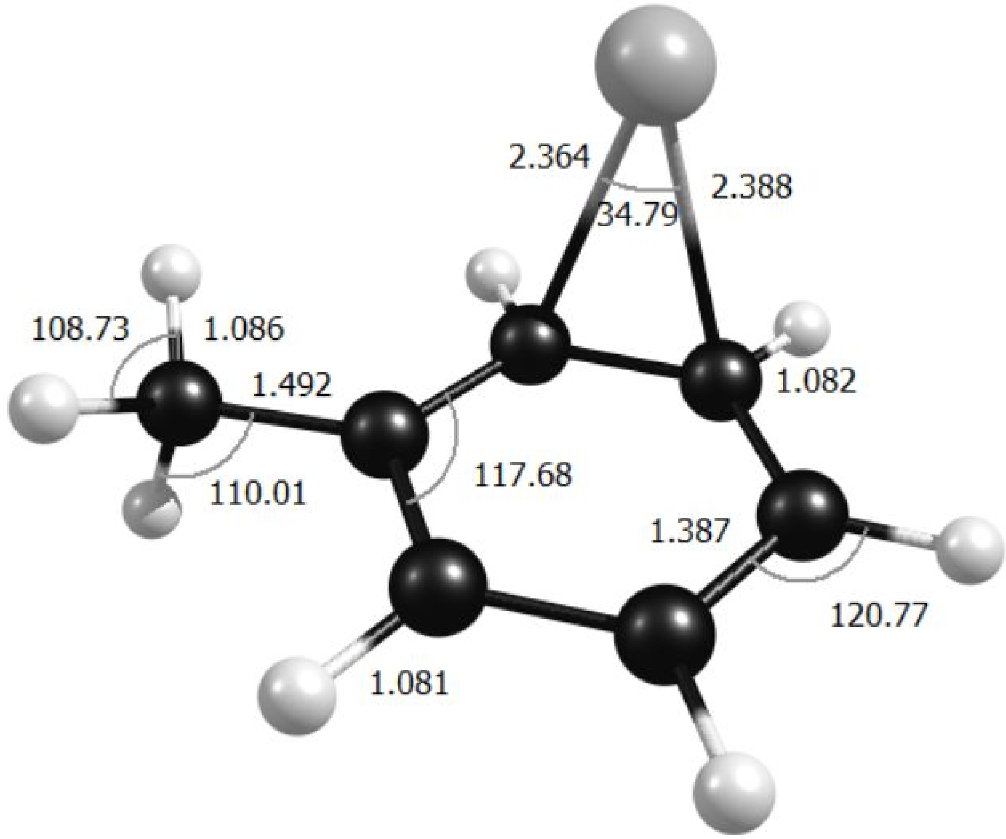

Coordinates:

Ag	1.380090000	-0.660308000	-0.023861000
H	-0.606220000	-0.997652000	1.889939000
C	-2.484370000	-1.563666000	-0.006519000
H	-2.047349000	-2.431810000	0.477706000
H	-2.711773000	-1.817192000	-1.037758000
H	-3.435854000	-1.367168000	0.486525000
C	-1.604101000	-0.362445000	0.085377000
C	-1.746854000	0.710011000	-0.793630000
C	-0.977151000	1.860577000	-0.674343000
C	-0.029119000	1.980385000	0.330734000
C	0.134711000	0.935001000	1.242966000
C	-0.649201000	-0.242685000	1.114578000
H	-2.487392000	0.646494000	-1.578456000
H	-1.126055000	2.674728000	-1.367747000
H	0.553726000	2.882518000	0.435069000
H	0.776595000	1.056373000	2.105242000

Table S1: Relative energies (kcal/mol; absolute energies in Hartrees in parentheses) from our computations for Ag^+(benzene) and Ag^+(toluene).

	B3LYP<br>def2-TZVP	def2-QZVP	M06L<br>def2-TZVP	def2-QZVP
Ag^+(benzene)	0.00<br>(-379.034941)	0.00<br>(-379.053755)	0.00<br>(-379.053974)	0.00<br>(-379.07729)
Ag^+(toluene)				
Isomer 1	0.00<br>(-418.343998)	0.00<br>(-418.366098)	0.00<br>(-418.356490)	0.00<br>(-418.383053)
Isomer 2	0.83<br>(-418.342670)	0.83<br>(-418.364773)	0.46<br>(-418.355751)	0.45<br>(-418.382333)

Table S2 : Calculation of Ag^+(benzene) excited state energies and oscillator strengths using TD-DFT (B3LYP/def2-TZVP). The oscillator strength is shown as an f value. Involved transitions, descriptions of the molecular orbitals, and their relative contributions are shown for each excited state. The molecular orbital depictions of these transitions are shown in Figure 5 in the main manuscript.

Transition energy		Oscillator Strength	Lower Orbital	Excited Orbital	Contribution	Description
cm^{-1}	nm		HOMO - m	LUMO + n		
28,317	353.14	0.0735	1	0	0.69858	Benzene π → Silver s
42,239	236.75	0.0143	7	0	0.13047	Silver d → Silver s
			5	0	0.46662	Silver d → Silver s
			4	0	-0.21282	Silver d → Silver s
			2	0	0.10316	Benzene s → Silver s
			1	2	0.23218	Benzene π → Benzene π
			0	1	-0.38849	Benzene π → Benzene π
42,753	233.9	0.0152	7	0	0.39899	Silver d → Silver s
			5	0	0.12244	Silver d → Silver s
			4	0	-0.32189	Silver d → Silver s
			2	0	0.17045	Benzene s → Silver s
			1	2	-0.26090	Benzene π → Benzene π
			0	1	0.34875	Benzene π → Benzene π
43,178	231.6	0.0105	7	0	-0.26753	Silver d → Silver s
			5	0	0.47359	Silver d → Silver s
			2	0	-0.31963	Benzene s → Silver s
			1	2	-0.18399	Benzene π → Benzene π
			0	1	0.23519	Benzene π → Benzene π
44,587	224.28	0.0001	6	1	-0.46222	Silver d → Silver s
			3	1	0.52895	Benzene s → Silver s
44,865	222.89	0.0008	7	1	0.49624	Silver d → Silver s
			4	1	0.37601	Silver d → Silver s
			2	1	-0.30776	Benzene s → Silver s
45,102	221.72	0.003	8	1	0.69957	Silver d → Silver s
46,577	214.7	0.0029	1	1	0.50165	Benzene π → Benzene π
			0	2	-0.48674	Benzene π → Benzene π
50,787	196.9	0.1596	1	1	0.41757	Benzene π → Benzene π
			0	2	0.40337	Benzene π → Benzene π
			0	3	0.38469	Benzene π → Silver π(?)

Table S3 : Calculation of Ag^{+}(toluene) Isomer 1 excited state energies and oscillator strengths using TD-DFT (B3LYP/def2-TZVP). The oscillator strength is shown as an f value. Involved transitions, descriptions of the molecular orbitals, and their relative contributions are shown for each excited state.

Transition Energy		Oscillator Strength	Lower Orbital	Excited Orbital	Contribution	Description
cm^{-1}	nm		HOMO - m	LUMO + n		
24,838	402.60	0.0154	1	0	0.56834	Toluene π → Silver s
			0	0	-0.41445	Toluene π → Silver s
25,798	387.62	0.0871	1	0	0.41475	Toluene π → Silver s
			0	0	0.56525	Toluene π → Silver s
44,966	222.39	0.0475	1	1	0.30203	Toluene π → Toluene π
			1	2	-0.2773	Toluene π → Toluene π
			0	1	0.48139	Toluene π → Toluene π
			0	2	0.27611	Toluene π → Toluene π

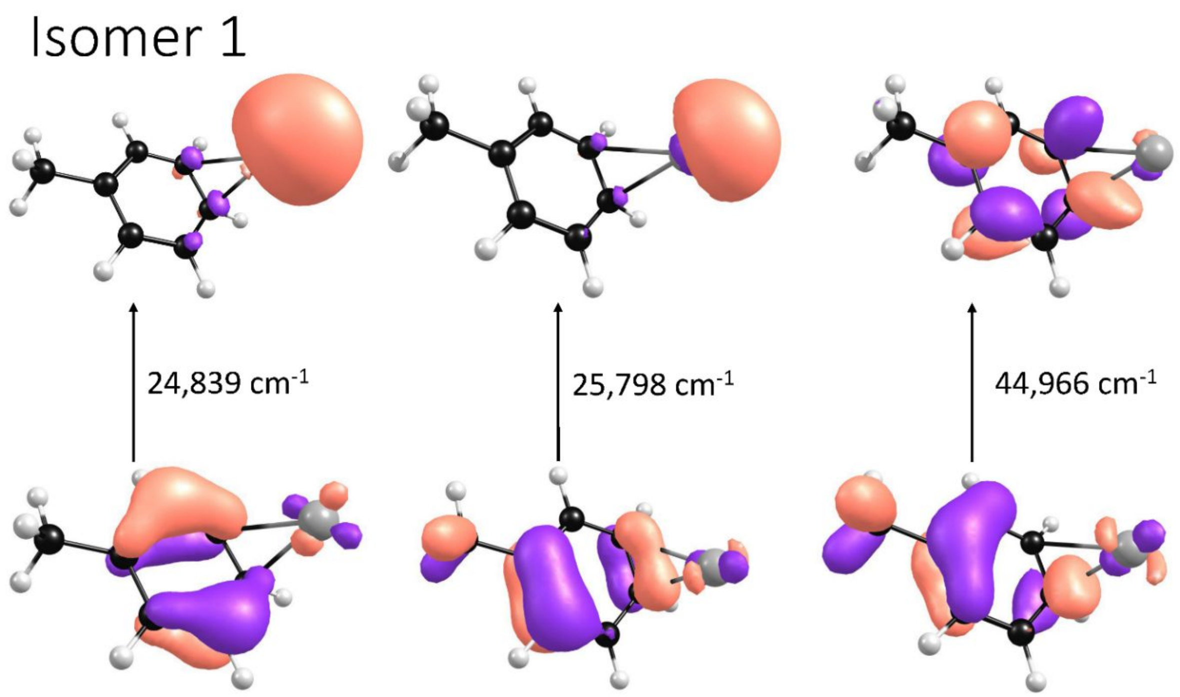

Figure S3. Molecular orbitals involved with each excited state transition for Ag^{+}(toluene) Isomer 1, calculated using TD-DFT (B3LYP/def2-TZVP). The two lower energy transitions appear to be of the form π(toluene) → *s* (Ag) charge transfer transitions. The higher energy transition appears to be of the form π(toluene) → π(toluene), i.e., a transition based on the toluene ligand.

Table S4 : Calculation of Ag^+(toluene) Isomer 2 excited state energies and oscillator strengths using TD-DFT (B3LYP/def2-TZVP). The oscillator strength is shown as an f value. Involved transitions, descriptions of the molecular orbitals, and their relative contributions are shown for each excited state.

Transition Energy		Oscillator Strength	Lower Orbital	Excited Orbital	Contribution	Description
cm^{-1}	nm		HOMO - m	LUMO + n		
27,838	359.21	0.0685	1	0	0.69864	Toluene π → Silver s
40,364	247.74	0.0212	10	0	-0.11399	Silver d → Silver s
			4	0	-0.34716	Silver d → Silver s
			3	0	0.3525	Toluene s → Silver s
			1	2	-0.18118	Toluene π → Toluene π
			0	1	0.44157	Toluene π → Toluene π
40,828	244.93	0.0244	7	0	-0.19552	Silver d → Silver s
			6	0	-0.13271	Silver d → Silver s
			4	0	0.17908	Silver d → Silver s
			3	0	-0.30866	Toluene s → Silver s
			2	0	-0.34181	Toluene s → Silver s
			1	2	-0.20311	Toluene π → Toluene π
			0	1	0.37381	Toluene π → Toluene π
40,995	243.93	0.0093	8	0	-0.16275	Silver d → Silver s
			7	0	0.18724	Silver d → Silver s
			6	0	-0.16623	Silver d → Silver s
			5	0	-0.19403	Silver d/methyl s → Silver s
			4	0	0.13446	Silver d → Silver s
			3	0	-0.25011	Toluene s → Silver s
			2	0	0.47925	Toluene s → Silver s
			1	2	-0.11693	Toluene π → Toluene π
			0	1	0.20314	Toluene π → Toluene π

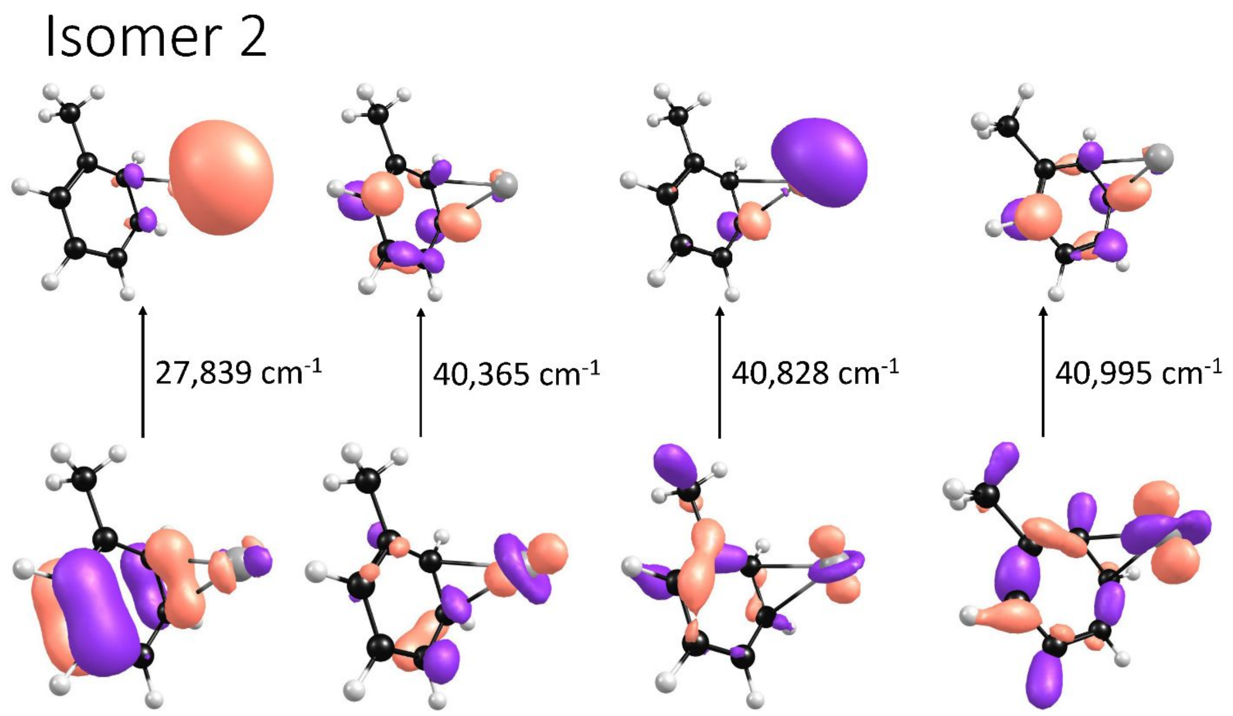

Figure S4. Molecular orbitals involved with each excited state transition for Ag^{+}(toluene) Isomer 2, calculated using TD-DFT (B3LYP/def2-TZVP). The lower energy transition appears to be of the form π(toluene) $\rightarrow$ *s* (Ag), i.e., a charge transfer transition. The higher energy transition appears to be of the form *d* (Ag^{+}) $\rightarrow$ *s* (Ag^{+}), i.e., the chromophore is based on the silver atomic ion.
